# Medical Applications of Nonadditive Entropies †

**DOI:** 10.3390/e25040578

**Published:** 2023-03-28

**Authors:** Constantino Tsallis, Roman Pasechnik

**Affiliations:** 1 Centro Brasileiro de Pesquisas Fisicas and National Institute of Science and Technology of Complex Systems, Rua Xavier Sigaud 150, Rio de Janeiro 22290-180, RJ, Brazil; 2 Santa Fe Institute, 1399 Hyde Park Road, Santa Fe, NM 87501, USA; 3 Complexity Science Hub Vienna, Josefstädter Strasse 39, 1080 Vienna, Austria; 4 Department of Physics, Lund University, Sölvegatan 14A, SE-22362 Lund, Sweden

**Keywords:** medical applications, nonadditive entropies, nonextensive statistical mechanics, image and signal processing

## Abstract

The Boltzmann–Gibbs additive entropy $S_{BG}=-k\sum_{i}p_{i}\ln p_{i}$ and associated statistical mechanics were generalized in 1988 into nonadditive entropy $S_{q}=k\frac{1-\sum_{i}p_{i}^{q}}{q-1}$ and nonextensive statistical mechanics, respectively. Since then, a plethora of medical applications have emerged. In the present review, we illustrate them by briefly presenting image and signal processings, tissue radiation responses, and modeling of disease kinetics, such as for the COVID-19 pandemic.

## 1. Introduction

Throughout the history of the sciences, medical applications have emerged within very diverse disciplines. In particular, physics has provided all kinds of such applications, including X-rays, magnetic resonances, optical fibers, laser instruments, thermometers, pressure devices, and radio therapies, among many others. In recent decades, new applications have emerged from statistical mechanics, one of the pillars of contemporary physics, together with electromagnetism, classical and quantum mechanics, and others. The processing of various medical images and signals, such as electroencephalograms (EEG), magnetoencephalograms (MEG), as well as other important procedures, have benefited, both in precision and in speed, from the thermostatistical concept of entropy, which, together with the concept of energy, provide the basis of classical thermodynamics [1]. More precisely, the 1988 proposal [2] of so-called nonadditive entropies as a basis to generalize the traditional Boltzmann–Gibbs (BG) theory led to many useful medical applications. We review here selected examples.

BG statistical mechanics is constructed upon the following Boltzmann–Gibbs–von Neumann–Shannon entropic functional:

(1) $$S_{BG}=-k\sum_{i=1}^{W}p_{i}\ln p_{i}\;\;\left(\sum_{i=1}^{W}p_{i}=1\right)\;,$$

where *k* is a conventional positive constant chosen once for ever (typically $k=k_{B}$ in physics, and k=1 in computational sciences). Its maximal value occurs for equal probabilities, i.e., $p_{i}=1/W\;,\forall i$, and is given by

(2) $$S_{BG}=k\ln W\;,$$

carved on the tombstone of Ludwig Boltzmann in Vienna. This relation constitutes an inspired connection between the macroscopic and the microscopic descriptions of real systems. The entropy (1) is *additive* [3]. Indeed, if *A* and *B* are two *probabilistically independent* systems (i.e., $p_{ij}^{A+B}=p_{i}^{A}p_{j}^{B}\;,\forall \left(i,j\right)$), we straightforwardly verify that

(3) $$S_{BG}\left(A+B\right)=S_{BG}\left(A\right)+S_{BG}\left(B\right)\;.$$

In addition, for a system in thermodynamical equilibrium with a thermostat at temperature *T*, the distribution which optimizes $S_{BG}$ is given by the celebrated BG weight

(4) $$p_{i}=\frac{e^{-\beta E_{i}}}{\sum_{j=1}^{W}e^{-\beta E_{j}}}\;,$$

where β=1/kT and $\left\{E_{i}\right\}$ are the possible energies of the system.

In 1988, a generalization of this theory was proposed [2] on the basis of the entropic functional

(5) $$S_{q}=k\frac{1-\sum_{i=1}^{W}p_{i}^{q}}{q-1}\;\;\left(q\in R;S_{1}=S_{BG}\right)\;.$$

Its maximal value is given by the generalization of Equation (2), namely

(6) $$S_{BG}=k\frac{W^{1-q}-1}{1-q}\equiv k\ln_{q}W\;.$$

Equation (3) is generalized as follows:

(7) $$\frac{S_{q}\left(A+B\right)}{k}=\frac{S_{q}\left(A\right)}{k}+\frac{S_{q}\left(B\right)}{k}+\left(1-q\right)\frac{S_{q}\left(A\right)}{k}\frac{S_{q}\left(B\right)}{k}\;,$$

hence

(8) $$S_{q}\left(A+B\right)=S_{q}\left(A\right)+S_{q}\left(B\right)+\frac{1-q}{k}S_{q}\left(A\right)S_{q}\left(B\right)\;.$$

And Equation (4) is generalized into

(9) $$p_{i}=\frac{e_{q}^{-\beta_{q}\left(E_{i}-\mu_{q}\right)}}{\sum_{j=1}^{W}e_{q}^{-\beta_{q}\left(E_{j}-\mu_{q}\right)}}\;,$$

where $\mu_{q}$ plays the role of a chemical potential, and $e_{q}\left(x\right)$ is the inverse function of $\ln_{q}x$, i.e.,

(10) $$e_{q}^{x}\equiv {\left[1+\left(1-q\right)x\right]}_{+}^{\frac{1}{1-q}}\;,$$

${\left[\ldots \right]}_{+}$ being equal to […] if […]>0 and zero otherwise.

Details related to this *q*-generalized statistical mechanics, currently referred to as *nonextensive statistical mechanics*, are available at [4,5], and a full bibliography is available at [6].

## 2. Medical Applications

The concept of entropy has more than once been useful in connection with medical applications (see, for instance, [7,8,9]). In particular, nonadditive entropies have been extensively used in image and signal processing in order to improve speed and clarity. Illustrative examples are provided here, as well as applications in tissue radiation response.

### 2.1. Image Processing

**Figure 1:**
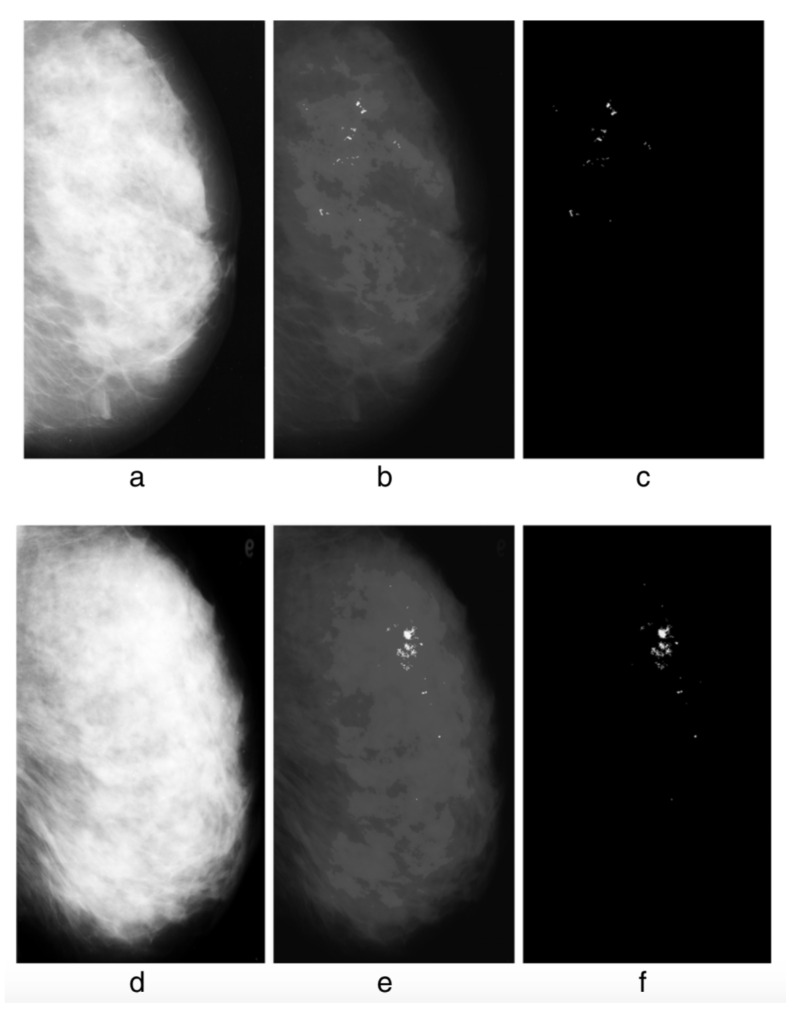
Without *q*-entropy enhancement with q≠1, detection of microcalcifications is limited: 80.21% Tps (true positives) with 8.1 Fps (false positives), whereas upon introduction of the *q*-entropy, the results surge to 96.55% Tps with 0.4 Fps. Detection results from the experiment: (**a**) mdb236, (**b**) output with the Mcs enhanced, (**c**) output with the Mcs extracted; (**d**) mdb216, (**e**) output with the Mcs enhanced, (**f**) output with the Mcs extracted. From [10].

The detection of possible pathological microcalcifications as revealed in mammograms can be improved by using *q*-entropy with q≠1 [10] (see Figure 1).

**Figure 2:**
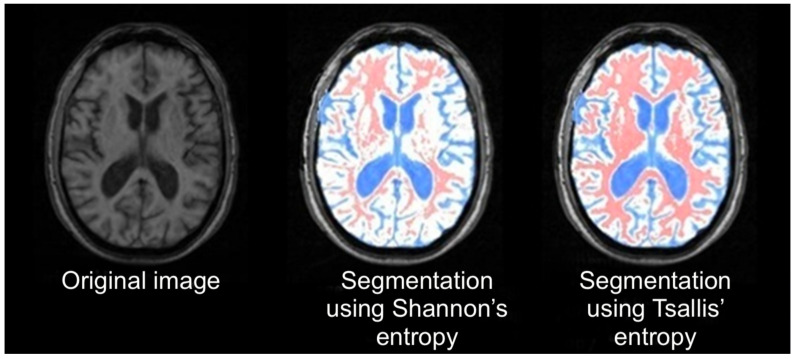
Image segmentation using Shannon (q=1) and *q*-entropy with q≠1. From [11].

Brain tissue segmentation using *q*-entropy improves the diagnosis of multiple sclerosis in magnetic resonance images [11] (see Figure 2).

**Figure 3:**
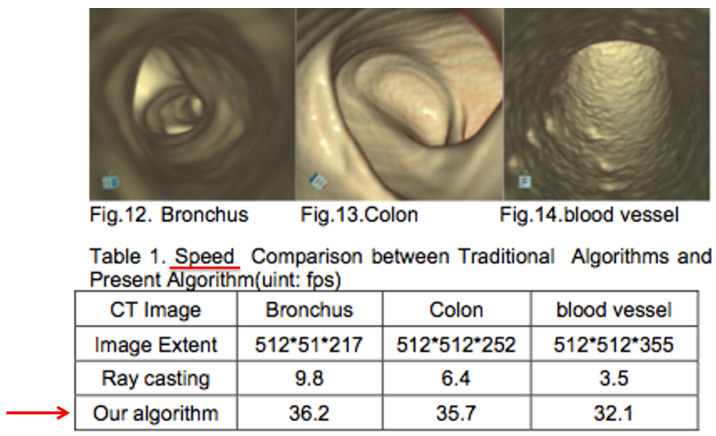
Detailed images of bronchus, colon, and blood vessel. From [12].

Detailed images of bronchus, colon, and blood vesse (Figure 3).

**Figure 4:**
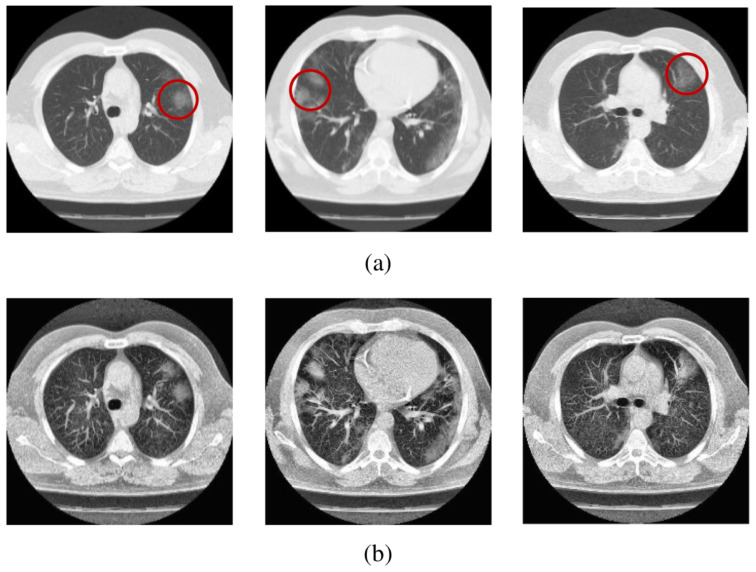
Sample scans from the dataset before and after enhancement showing infected lungs. (**a**) Original computer tomography scans, with red circles highlighting some regions where fibrosis can be seen; (**b**) enhanced computer tomography scans using q=0.5. Further details can be seen in [13].

The images of computer tomography scans revealing fibrosis due to COVID-19 can be improved by incorporating into the algorithm the *q*-entropy with q=0.5 [13] (see Figure 4).

### 2.2. Signal Processing

As a critical element of cardiovascular research, in the examination of heart health an electrocardiogram (ECG) represents a record of cardiac electrical activity whose sophisticated analysis is highly relevant for diagnosing and preventing cardiovascular diseases. The latter typically requires considerable human resources and expertise. To this end, many powerful automated techniques and methodologies for ECG signal analysis have been reported to date in the literature (see e.g. [14,15]; for recent reviews, see [16,17] and references therein). Most advanced methods, in particular, utilise modern artificial intelligence approaches enabling rapid human-like interpretation of the ECG recordings capable of recognising subtle patterns and details in the ECG signals typically inaccessible by human interpreters [18,19,20]. Such methods make ECG signal analysis a powerful, non-invasive means of biomarking.

Another important method for monitoring and examining patient health is based on electroencephalogram (EEG) recordings [21,22]. This method is particularly focused on controlling disruptions in the functionality of neurons inside the brain, such as seizures in epilepsy [23]. Existing conventional treatments for epilepsy cannot be efficiently applied in the case of successive seizures, which are widespread and account for about 30% of epilepsy patients [24]. A comprehensive visual analysis of EEG recordings by doctors is cumbersome as it takes too much time and can be subjective and prone to human error. Hence, an automated seizure detection approach is required to accelerate the analysis of EEG recordings and obtain more accurate predictions [25,26,27,28].

Both ECG and EEG recordings are important ingredients of so-called medical time series representing recorded electronic health datasets containing important information on certain aspects of a patient’s health recorded over a given period of patient care, or over the course of a patient’s entire lifetime [29]. Generic medical (or clinical) time series may capture genetic and lifestyle health risks, indicate the emergence of possible diseases, and contain information about the time and stage of diagnosis, as well as about the development of treatment plans. A detailed and reliable analysis of medical time series is essential for understanding clinical trajectories and progression in a wide range of diseases, such as cancer, Alzheimer’s or cardiovascular disease, etc., as well as for enabling precise forecasting of disease trajectories, correct and timely diagnosis, and the development of appropriate treatment procedures [30].

**Figure 5:**
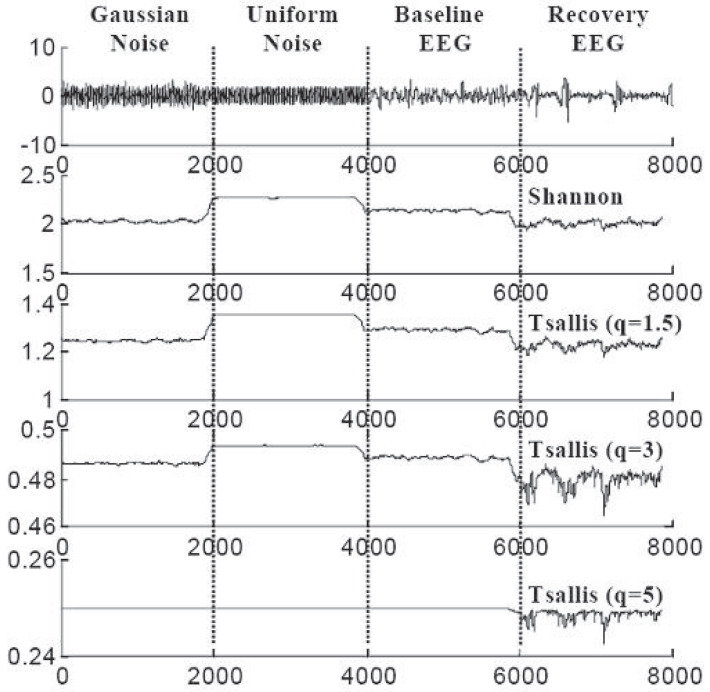
The goal is to distinguish between signals with different probability distributions, and between EEG recordings for different physiological conditions. The optimal is achieved for q≃3. Further details can be seen in [31].

**Figure 6:**
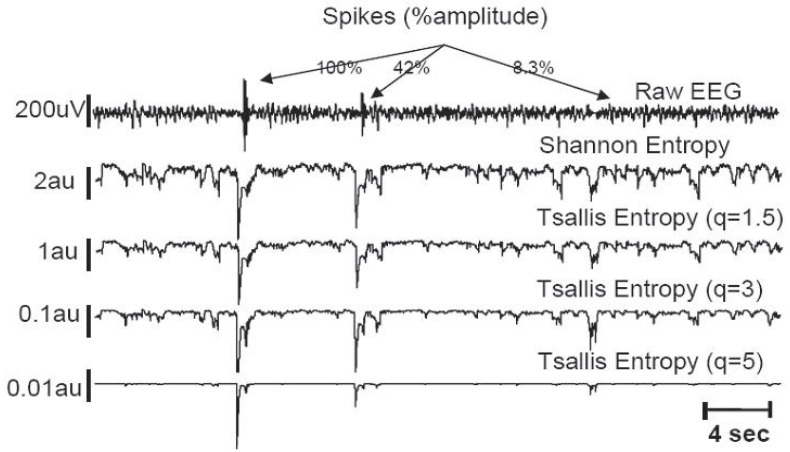
The goal is to detect the existence of three (artificially introduced) spikes which corrupt the raw EEG. Even small spikes become detectable after processing with q≥3. Further details can be seen in [31].

Signal processing of the EEG for direct medical use has been proposed for brain injury following serious events, such as cardiac arrest or asphyxia [31]. Typical results are indicated in Figure 5 (where the highest sensitivity of the recovery EEG is achieved for q≃3) and Figure 6 (where artificial low-amplitude spikes become detectable after (entropic) processing with q≥3). Further biomedical applications are described in [32,33,34,35,36,37,38,39,40,41,42,43,44].

**Figure 7:**
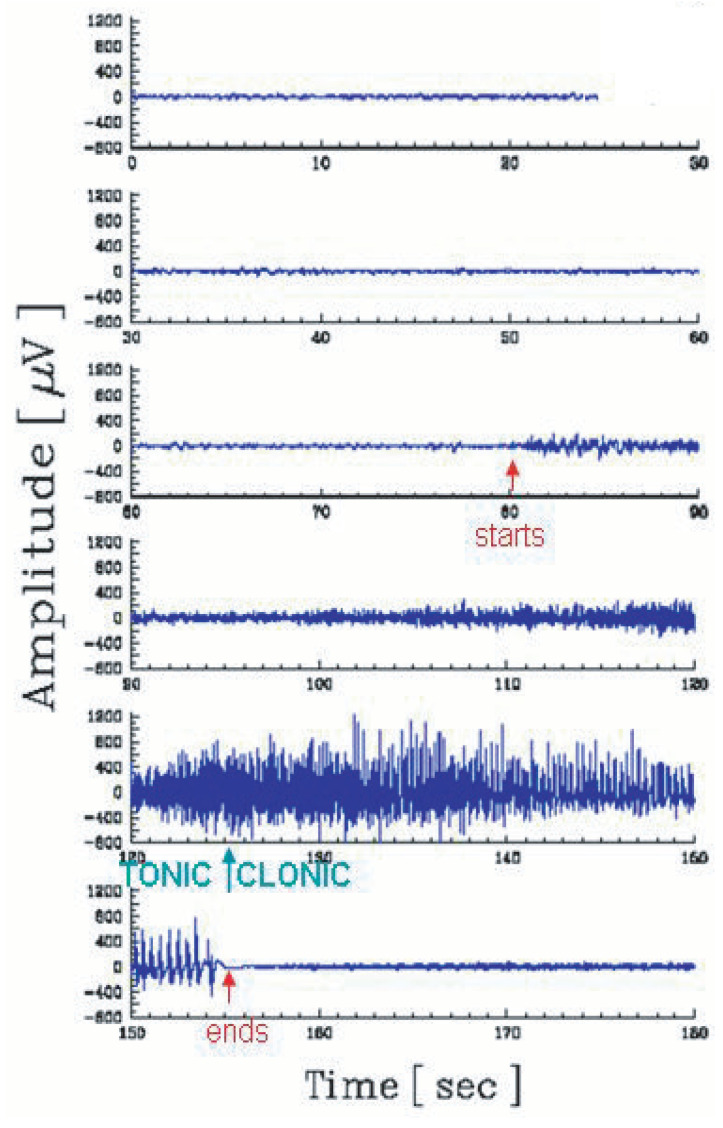
Electroencephalogram (including the contribution of muscular activity) during an epileptic crisis, which starts at 80 s, and ends at 155 s. By direct inspection of the EEG, it is virtually impossible to detect the (clinically dramatic) transition (at 125 s) between the tonic stage and the clonic stage of the patient. Further details can be seen in [42].

**Figure 8:**
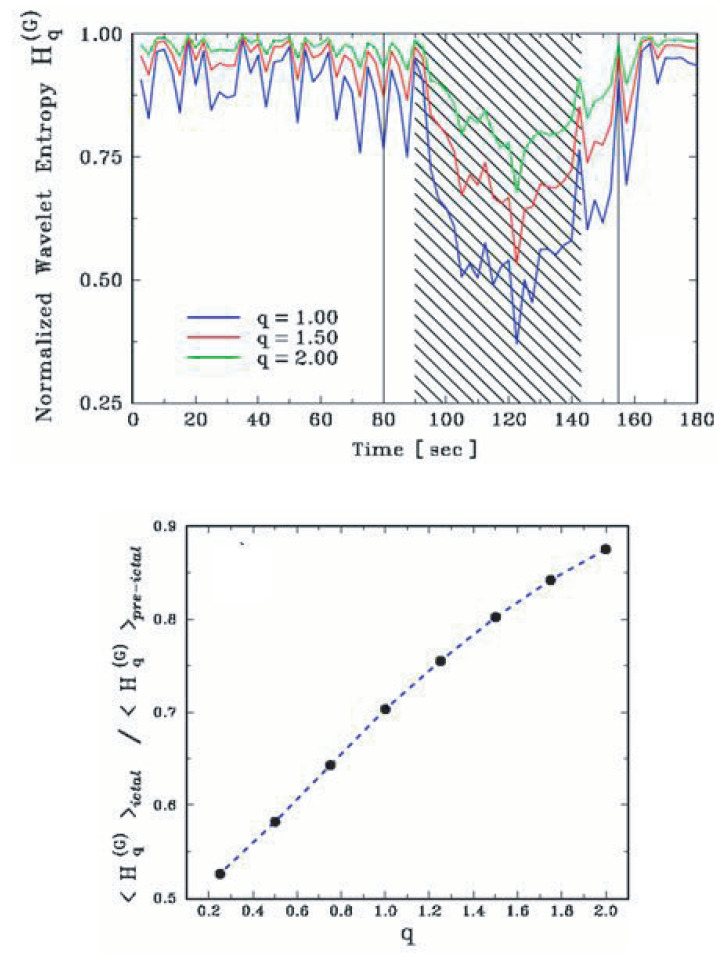
(**Top panel**) After processing (of the EEG signal), which includes the use of the entropic functional $S_{q}$, the precise location of the tonic–clonic transition becomes very visible. (**Bottom panel**) The effect is even more pronounced for values of *q* going below unity. Further details can be seen in [42].

The analysis of the tonic–clonic transition of some types of epilepsy provides a typical illustration [42]. The EEG during a crisis can be seen in Figure 7. At time 125 s, a clinically dramatic transition occurs with the patient. However, nothing special can be seen in the direct EEG at that moment. In contrast, as we verify in Figure 8, after appropriate processing, the tonic–clonic transition becomes clearly visible. The discrimination becomes even stronger if q<1 is used. If no specialized medical agents are present at the precise moment of the crisis of the patient, the existence of such a neat peak makes possible the automatic start of computer-controlled administration of appropriate drugs during the emergency.

The use of such algorithms is expected to enable improved analyses in mild cognitive impairment, vascular dementia, Lewy body dementia, major depression, dementia associated with Parkinson’s disease, Pick’s disease, Huntington’s chorea, and progressive supranuclear palsy, among others.

### 2.3. Tissue Radiation Response

Radio therapies are frequently used to aid recovery from serious diseases, such as cancer. The application of such medical procedures is, however, quite difficult. Indeed, healthy cells can be attacked together with sick ones.

**Figure 9:**
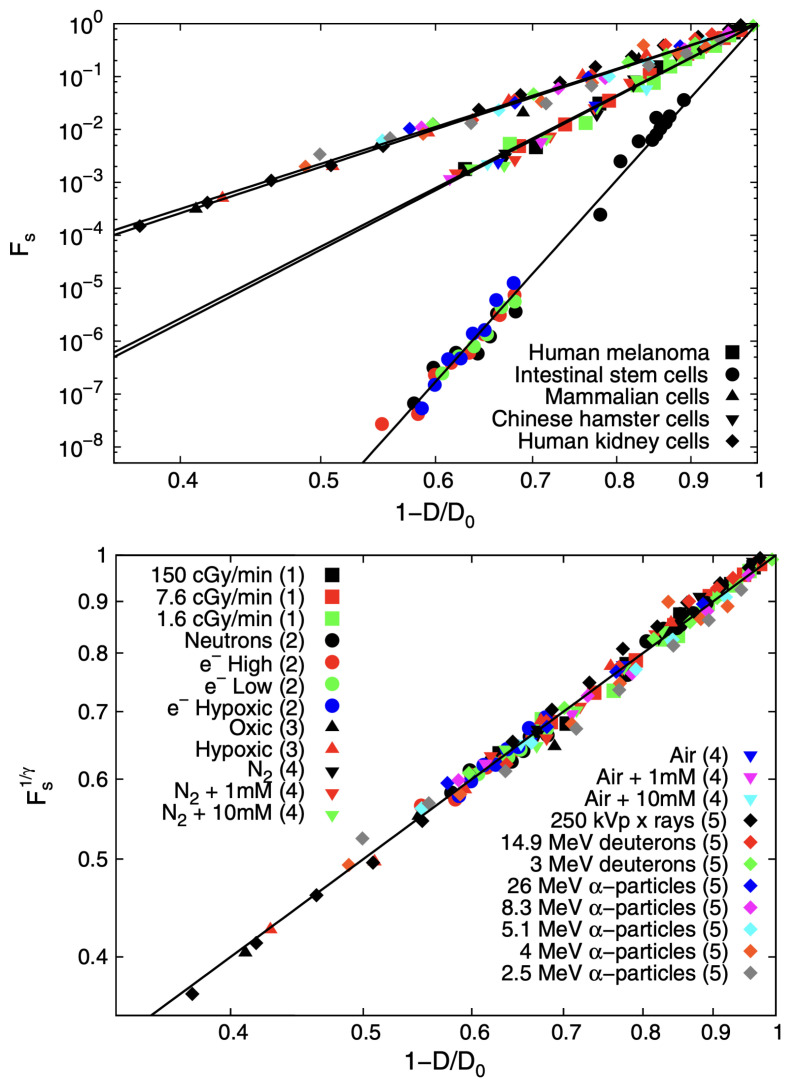
(**Top panel**) Survival fraction $F_{s}$ as a function of the rescaled radiation dose $\left(1-D/D_{0}\right)$ for different tissues: intestinal stem cells, Chinese hamster cells, human melanoma, human kidney cells, and cultured mammalian cells under different irradiation conditions. Various shapes represent tissues, whereas each color highlights different radiation conditions. Five solid lines represent fitting to experimental data. (**Bottom panel**) Collapsed survival fractions $F_{s}$ for different tissues with γ≡(2−q)/(1−q): intestinal stem cells (γ≃30.5), Chinese hamster cells (γ≃14.0), human melanoma (γ≃14.0), human kidney cells (γ≃8.9), and cultured mammalian cells (γ≃8.9) under different irradiation conditions. Further details can be seen in [45].

**Figure 10:**
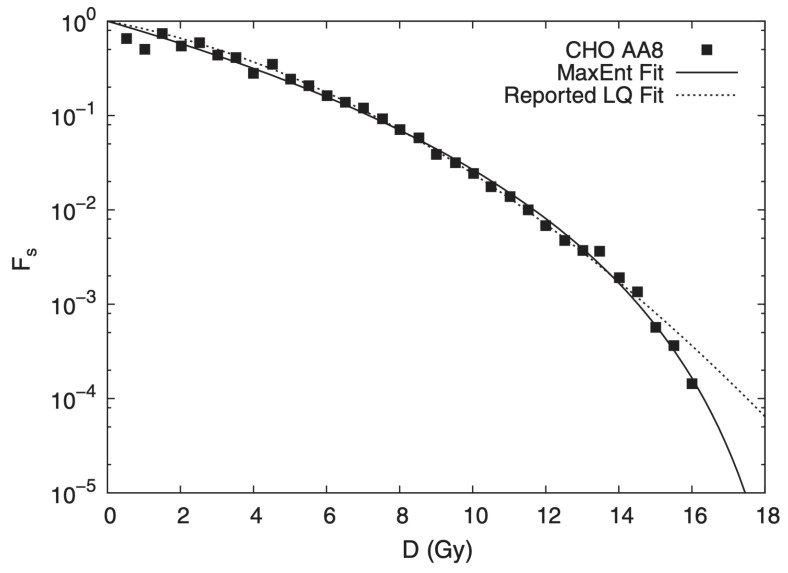
Comparison between the current linear-quadratic (LQ) BG-like exponential model best fit and the present *q*-exponential model fitted to γ=5.0±0.04 and $D_{0}=\left(19.4\pm 0.4\right)\;Gy$ for the cell line *CHO AA8* under 250 k-Vp x rays. Further details can be seen in [45].

From *q*-statistical arguments, it was obtained [45] that the *cell survival fraction* $F_{s}$ is given by the *q*-exponential form $F_{s}={\left(1-D/D_{0}\right)}^{\gamma }$, where *D* is the applied dose, $D_{0}$ is the minimal annihilation dose, and γ≡(2−q)/(1−q). Figure 9 shows the validation of this expression with experimental data obtained for five different classes of cells; moreover, a universal curve can be established through appropriate collapse. The superiority of the *D*-dependence of $F_{s}$ is illustrated in Figure 10. Indeed, the use of the current linear-quadratic (LQ) exponential function (of the BG type) yields, for extreme values of *D* (such as D=16Gy), a value for $F_{s}$ which can be erroneous by a dangerous factor larger than two in the survival fraction. Such an overdose can be fatal for healthy cells.

## 3. Modeling of Disease Kinetics

The complexity of the propagation of diseases within a population makes these phenomena strong candidates for the application of *q*-statistics. We mention here two such applications for COVID-19, one of them being purely descriptive [46], the other one entering into the dynamics of an epidemic or pandemic [47].

**Figure 11:**
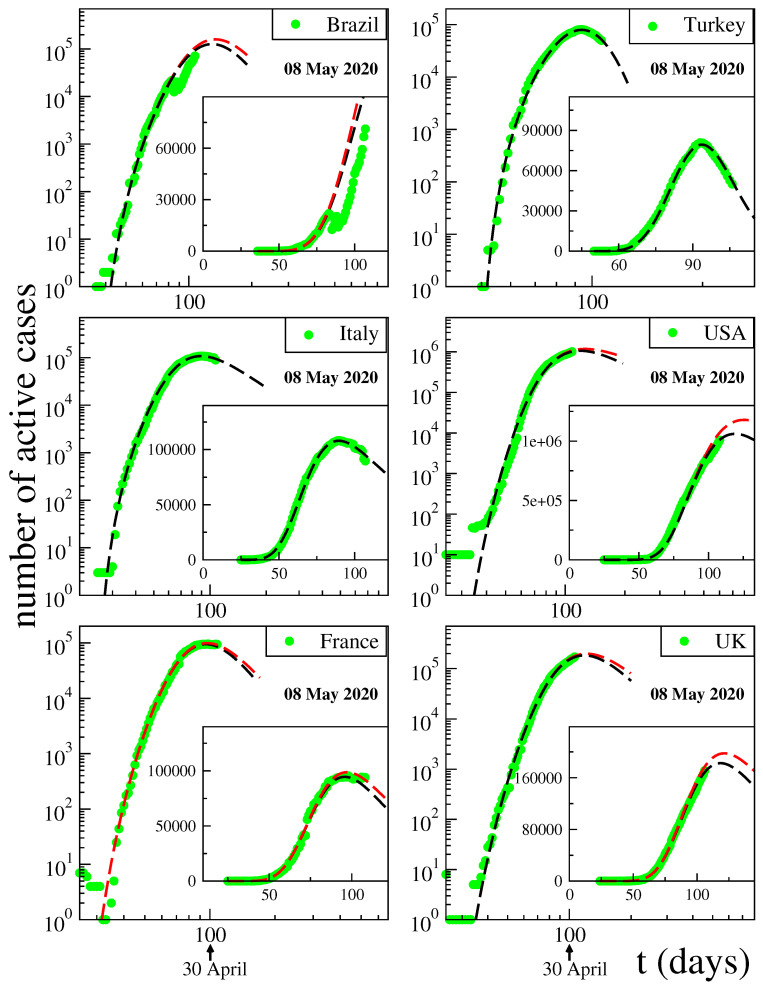
Pandemic evolution of COVID-19. Further details can be seen in [46].

Let us briefly review the quite successful description advanced in [46] for the behaviour of total cases and fatality curves. We will concentrate here on analysis of the active cases and deaths *per day*. The inspection of public data, such as [48] (updated on a daily basis), in particular, of the time evolution of the number *N* of active cases (surely a lower bound of the unknown actual numbers) showed a rather intriguing similarity with the distributions of the volumes of stocks. Along these lines, we adopt the following functional form for each country or region:

(11) $$N=C{\left(t-t_{0}\right)}^{\alpha }e_{q}^{-\beta \;{\left(t-t_{0}\right)}^{\gamma }}=\frac{C{\left(t-t_{0}\right)}^{\alpha }}{{\left[1+\left(q-1\right)\beta \;{\left(t-t_{0}\right)}^{\gamma }\right]}^{1/(q-1)}}\;,$$

with C>0;α>0;β>0;γ>0, q>1 and $t_{0}\geq 0$. The constant $t_{0}$ indicates the first day of appearance of the epidemic in that particular country (or region). The normalising constant *C* reflects the total population of that particular country. For α=0, if γ=1, we recover the standard *q*-exponential expression; if γ=2, it is currently referred to in the literature as *q*-Gaussian; for other values of γ, it is referred to as stretched *q*-exponential. Through the inspection of the roles played by the four nontrivial parameters, namely (α,β,γ,q), it became rather transparent that (α,β) depend strongly on the epidemiological strategy implemented in that region, in addition to the biological behaviour of the coronavirus in that geographical climate. In contrast, the parameters (γ,q) appear to be fairly universal for COVID-19, mainly depending on the coronavirus itself. See Figure 11 for typical results in May 2020.

## 4. Final Remarks

The *q*-exponential function $e_{q}^{x}={\left[1+\left(1-q\right)x\right]}^{\frac{1}{1-q}}$ is asymptotically universal for x→0, i.e., it does not depend on *q* for |x|→0. In other words, in such a limit, we are allowed to simply replace it by $e^{x}$. But the discrepancy becomes dramatic as soon as |x| grows. Since the index *q* generically reflects non-local space and/or time correlations which are virtually always present in relevant properties of complex systems, it is no surprise that nontrivial values of *q* can be usefully adapted in very many medical applications. Indeed, human beings, and living organisms in general, are notoriously complex systems and their properties frequently differ from the typical ones verified in simple systems, such as those in, say, the standard thermal equilibrium. The practicality of *q*-statistical concepts with regard to the usual Boltzmann–Gibbs concepts has been illustrated in the present review with examples including the processing of medical images (e.g., mammograms, computer tomography, magnetic resonance) and signals (e.g., EEG, MEG), tissue response to radiation, and the modeling of pandemic disease kinetics, such as for COVID-19. It appears that the door is open for the development of new and more precise procedures and algorithms that will be beneficial to human health.
